# Association Between Aneurysmal Haemodynamics and Device Microstructural Characteristics After Flow-Diversion Treatments With Dual Stents of Different Sizes: A Numerical Study

**DOI:** 10.3389/fphys.2021.663668

**Published:** 2021-05-25

**Authors:** Mingzi Zhang, Simon Tupin, Yujie Li, Makoto Ohta

**Affiliations:** ^1^Biomedical Flow Dynamics Laboratory, Institute of Fluid Science, Tohoku University, Sendai, Japan; ^2^ElyTMaX, CNRS—Université de Lyon—Tohoku University, International Joint Unit, Tohoku University, Sendai, Japan

**Keywords:** computational fluid dynamics, intracranial aneurysm, flow-diverting stent, dual-stent treatment, device sizing effect

## Abstract

**Objectives:**

Treating intracranial aneurysms with flow-diverting stents sometimes requires deployment of a second device. Herein we quantify the sizing effects of devices in dual-stent treatments upon the final stent microstructure and the post-treatment aneurysmal haemodynamics.

**Methods:**

Fifteen sidewall ICA aneurysm geometries were included. Using a virtual stenting technique, we implanted either one or two stents for each aneurysm treatment considered, with each stent specified as one of two different sizes, yielding a total of two single-stent and fouir dual-stent treatment scenarios for each aneurysm. Three stent microstructural parameters and nine aneurysmal haemodynamic parameters were quantified and systematically compared across the 90 treatment scenarios.

**Results:**

Deployment of a second stent further reduced the aneurysmal inflow rate (IR) and energy loss (EL) by, respectively, 14 ± 11% (*p* = 0.001) and 9 ± 12% (*p* = 0.056), relative to the untreated condition. Sizing effects of the earlier-deployed stent led to largest differences of 6.9% for the final IR reduction and 11.1% for the EL, whereas sizing effects from the later-deployed stent were minor (≤2.1%). The change in stent pore size was the only microstructural parameter demonstrating a strong correlation with the reduction in the post-treatment aneurysmal haemodynamics, in terms of the IR (*r* = 0.50, *p <* 0.001) and pressure drop (*r* = 0.63, *p <* 0.001).

**Conclusion:**

Size of the earlier-deployed stent has substantial effects on the final haemodynamic outcomes after dual-stent treatment. The average pore size of stent wires at the aneurysm orifice shows promise as a potential index for predicting the efficacy of flow-diversion treatments.

## Introduction

Flow-diverting (FD) stent intervention has become a common endovascular therapy, especially for the treatment of fusiform and wide-necked intracranial aneurysms (IAs) (Nelson et al., 2011; Becske et al., 2013). By placement of a metallic tubular mesh across the aneurysm orifice, the implanted device predominantly excludes the aneurysm from the parent arterial circulation, so as to induce blood clotting and finally a thrombotic occlusion of the aneurysm (Chung and Cebral, 2015).

Complete aneurysm occlusion is closely associated with sufficient flow diversion produced by the stent wires that partially cover the aneurysm orifice (Cebral et al., 2014). For this reason, implantation of an additional stent, either planned in the original intervention or added in a subsequent procedure, is sometimes required to compensate for inadequate flow diversion produced by the first stent alone (Zanaty et al., 2014).

Treatments with dual stents of different diameters may result in distinct haemodynamic outcomes, due to the variety of wire configurations following multi-stent implantation (Shapiro et al., 2014; Kole et al., 2019; Zhang et al., 2020). However, few studies have investigated such variations in stent morphology and the subsequent IA haemodynamics; nor has the correlation between them been established.

To fill in the gaps, we quantified the changes in stent morphology and IA haemodynamics in a cohort of 15 patient aneurysms, before and after dual-stent treatment with devices of different sizes, with reference to single-stent treatment, seeking (i) to establish the correlation between the stent microstructural characteristics (porosity, etc.) and the resulting IA haemodynamics (inflow rate, etc.); and (ii) to examine whether choice of the stents would have determinative effects on the final flow-diversion efficacy after dual-stent implantation.

## Materials and Methods

### Patient Selection

To enable cross-comparison of the present analyses with published studies, we accessed the open-source aneurysm imaging repository *Aneurisk* (Aneurisk-Team, 2012), and screened all aneurysm geometries (*n* = 103) in accordance with the following exclusion criteria: (i) aneurysms located in arteries other than the ICA segment; (ii) aneurysms with draining arteries or blebs developed on the wall; (iii) aneurysms initiated at an arterial bifurcation; (iv) multiple aneurysms developed adjacent to each other; and (v) aneurysms deemed unsuitable to be treated with FD stents in accordance with the Instructions for Use (IFU) (United States Food and Drug Administration, 2011) of the Pipeline Flex Embolisation device (PED, Medtronic, United States).

We set these criteria to exclude cases where precise segmentation of the vasculature may be difficult; and to include only aneurysms with similar parent-arterial flow conditions for further haemodynamic and statistical analyses. Geometries of the included aneurysms were immediately acquired from the *Aneurisk* repository. Use of the data from the *Aneurisk* repository had been approved by the ethical committee of the Ca’ Granda Niguarda Hospital, Milano, Italy.

### Virtual FD Stent Intervention

For the included cases, we modeled flow-diversion treatments with the PED. Virtual deployment of the PED was realised through use of a previously reported and validated spring–mass method (Spranger et al., 2015; Peach et al., 2016; Zhang et al., 2017b). (See Supplementary Material 1 for a detailed description.) For the deployment of a single stent, the boundary constraint was only the vascular wall. For the deployment of a second stent, we first performed a surface fitting of the fully expanded nodes (i.e., intersections of stent wires) of the earlier-deployed stent using SolidWorks (Dassault Systemes, Paris, France), and then set the fitted surface (treated as a vascular lumen), along with the vascular wall, as the boundary constraints (see Supplementary Figure 1 for a schematic diagramme).

Given the large longitudinal variation of the parent-arterial diameter within each of the cases, a range of stent diameters could be selected based upon the treating clinicians’ judgements. We therefore investigated the single-stent treatment for each aneurysm geometry with two plausible device diameters determined following the IFU (United States Food and Drug Administration, 2011): one that would just *fit* the recipient artery, denoted “F”; the other being one size *larger* (with enhanced vascular apposition), denoted “L.” To model the plausible dual-stent treatments, we deployed FD stents at each of the two sizes, respectively, into each of the earlier-deployed ones, yielding a total of four dual-stent scenarios—“fit into fit (FinF),” “fit into larger (FinL),” “larger into fit (LinF),” and “larger into larger (LinL).”

### Microstructure Analysis of the Stent Wires

The local microstructure of stents was examined following the ISO standard for evaluation of cardiovascular implants (International Organization for Standardization, 2016), through use of MATLAB R2019b (Mathworks, Natick, MA, United States).

**FIGURE 1:**
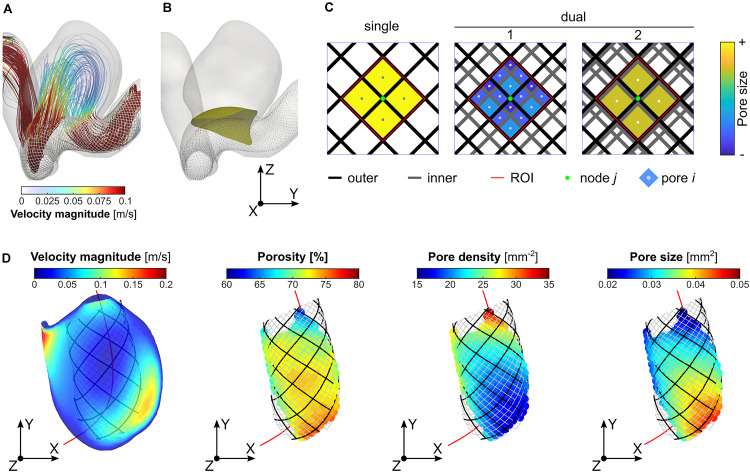
Determination of the region of interest (ROI) and calculation of the local porosity, pore density, and pore size: **(A)** visualisation of streamlines (Case ID: C0016), **(B)** illustration of stent “nodes” (i.e., intersections of stent wires) and a neck reference surface; **(C)** determination of the ROI (area within the dashed red lines) corresponding to a specific “node” (the central point in green) of the outer-layer stent, and measurement of the average local pore size; and **(D)** visualisation of velocity distribution on the reference neck surface, and plots of the three microstructural parameters on every stent “node” within the aneurysm orifice.

For each treatment scenario, we first obtained a series of images by projecting the stent wires onto the tangent planes at each “node” of the outer-layer stent (Figure 1B). From each projection, a region of interest (ROI) with four pores immediately surrounding the “node” was then extracted (Figure 1C). Based upon the ROI, the local porosity (φ), pore size (*S*_*p*_), and pore density (*D*_*p*_) were calculated as:

(1) $$\varphi =100\times \frac{\sum_{i=1}^{n}A_{i}}{A_{\text{ROI}}}$$

(2) Sp=∑i=1nAi2∑i=1nAi

(3) $$D_{\text{p}}=\frac{n}{A_{\text{ROI}}}$$

where *A*_*ROI*_ denotes the area of the ROI; *A_i_* refers to the area of the *i*^*th*^ pore; and *n* the number of pores within the ROI. Due to the variation in pore size within the neck plane, a weighted mean evaluation was determined for the pore size in order to give more importance to bigger pores, which have a greater influence on the flow resistance. For each of the three parameters, the mean and SD corresponding to the projections of nodes within a reference neck surface (determined on a patient-by-patient basis) were calculated and used for further analysis (Figure 1D).

### Blood Flow Simulations

The arterial wall and stent surface were assumed to be rigid, and a no-slip condition was imposed at these boundaries. To contrast the haemodynamic changes before and after the deployment of single or dual stents, we performed steady-state CFD simulations by setting a constant volumetric flowrate condition at the ICA inlet using a validated power-law estimate (Chnafa et al., 2018). At each outlet, a zero-pressure reference condition was applied. The density and viscosity of the blood were assumed to be 1,050 kg/m^3^ and 0.0035 Pa⋅s.

SnappyHexMesh embedded in the open-source CFD library OpenFOAM (Greenshields, 2019) was used to generate a predominantly hexahedral computational grid, with the maximal sizes of 0.4, 0.1, and 0.01 mm respective for the volume, aneurysm surface, and the FD stent surface. Across different cases, the total number of grid elements ranged from 0.5 to 18 million for the untreated, single-stent and dual-stent conditions. We obtained the post-treatment haemodynamics by solving the Navier–Stokes equations using the simpleFoam solver (OpenFOAM 7). The criteria of convergence were chosen as 10^–5^ for the normalised continuity, velocity, and pressure residuals. An integrated super-computation system (based on Fujitsu Server PRIMERGY RX2550M4) at the Institute of Fluid Science, Tohoku University was used, and the average simulation time for each case was around 6 h with 32 cores in parallel.

Iso-velocity surfaces, streamlines, and wall shear stress were first examined for each treatment scenario. To quantify the flow-diversion outcomes, we calculated the reductions in the IR, PD, EL, aVEL, aVOR, aWSS, mVEL, mVOR, and mWSS.

### Statistical Analysis

Independent two-sided *t*-tests were used to compare the changes in stent microstructure and IA haemodynamics between treatments with a single stent (*n* = 30) and with dual stents (*n* = 60). Correlations between changes in the stent microstructure and changes in the IA haemodynamics after dual-stent implantation were assessed by examining the Pearson correlation coefficient, *r*.

To investigate differences in the stent microstructure and IA haemodynamics after treatments with single stents of different sizes, paired-sample two-sided *t*-tests were performed (i.e., comparison between scenarios “F” and “L”). For dual-stent treatments: (i) Pearson χ^2^ tests were used to examine the sizing effects of the earlier-deployed stent; and (ii) paired-sample two-sided *t*-tests were used to compare the differences between the later-deployed stents of different sizes (i.e., comparisons between “FinF” and “LinF,” and between “FinL” and “LinL”).

The statistical analyses were carried out using MedCalc version 19.1 (Ostend, Belgium), supplemented with MATLAB R2019b. Throughout this work *p* < 0.05 was considered suggestive of statistical significance, while *p* < 0.005 was deemed to confirm statistical significance.

## Results

### Patient Characteristics

**FIGURE 2:**
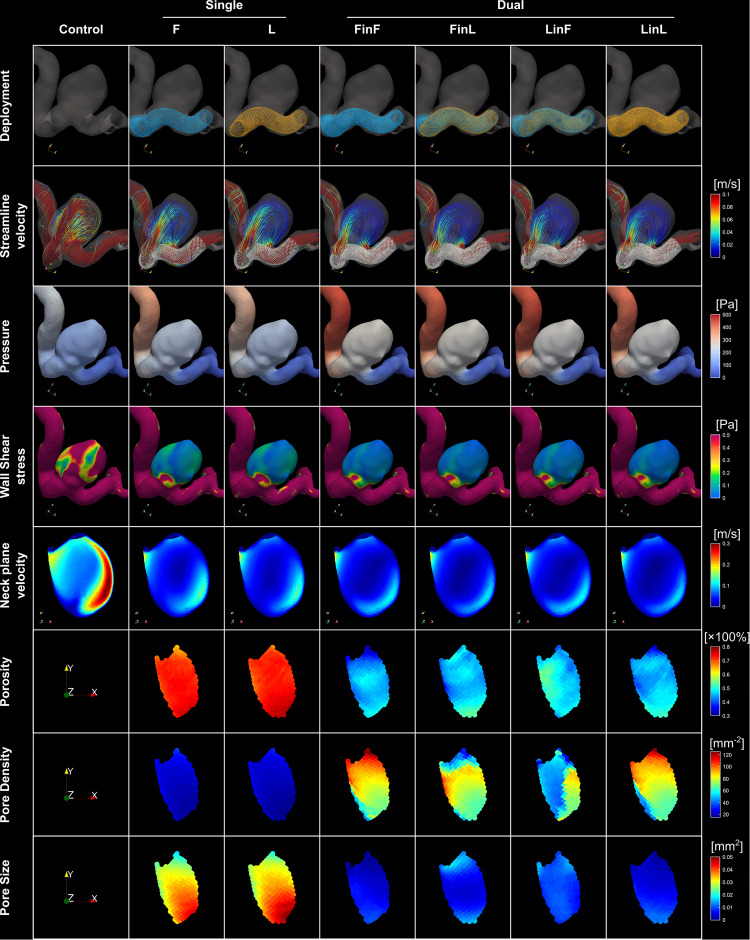
(i) Visualisation of stent structures corresponding to different treatment scenarios (*first row*); (ii) visualisation of streamlines, pressure and WSS distributions corresponding to each treatment mode (*rows two to four*); (iii) velocity distributions on the reference neck surface (*row five*); and (iv) distributions of the porosity, pore density, and pore size on stent nodes within the aneurysm orifice (*last three rows*) for a representative patient aneurysm (Case ID: C0016).

Fifteen geometries of aneurysms from 15 patients (female: 14, 93%), at mean age 50 years (range: 26–85) met the inclusion criteria. The included IAs had a mean aspect ratio of 1.27 (range: 0.64–1.76), a mean neck width of 5.98 mm (range: 2.61–9.34), and a mean dome-to-neck ratio of 1.27 (range: 0.83–1.77). The mean volumetric flowrate at the ICA inlet was 2.75 ± 1.30 mL/s. See Supplementary Table 1 for a summary of patient demographics, Supplementary Figure 2 for the IA morphology and stent structures after dual-stent implantation, and Figure 2 for the stent microstructural and IA haemodynamic analyses for a representative case.

### Stent Configurations and IA Haemodynamics After Single- or Dual-Stent Treatment

**FIGURE 3:**
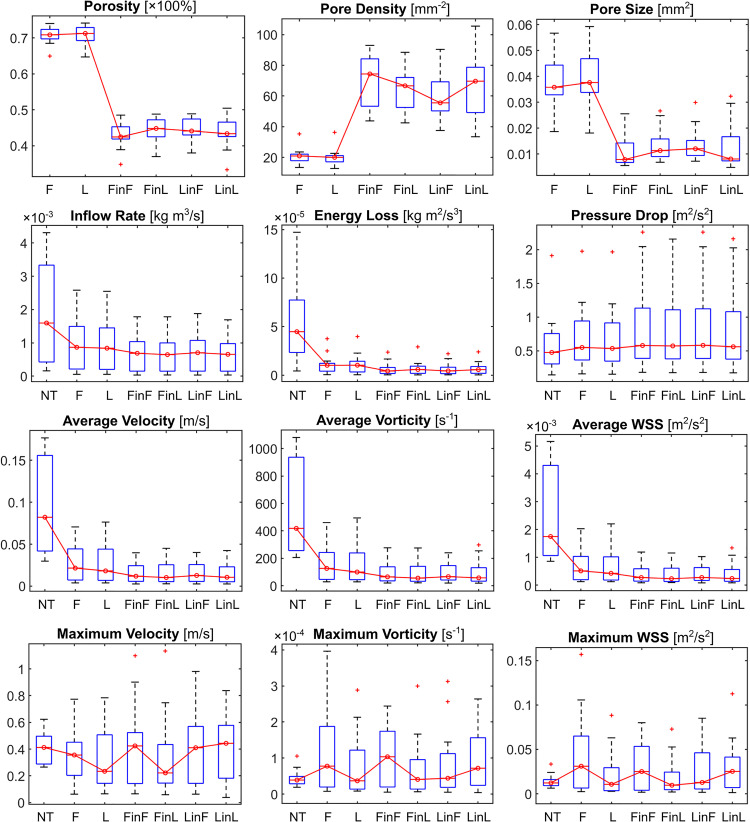
Boxplots of the stent microstructural and IA haemodynamic parameters corresponding to different treatment scenarios. NT: untreated condition; F: treatment with a single stent of “fit” size; L: treatment with a single stent of “larger” size; and FinL: dual-stent treatment with the later-deployed stent being of “fit” size and the earlier-deployed stent being of “larger” size. The same naming convention applies to the remaining treatment scenarios: FinF, LinF, and LinL.

Across the 15 cases, the average pore density, porosity, and pore size at the aneurysm orifice after single-stent treatment were, respectively, 20.3 ± 5.2 mm^–2^, 71 ± 2%, and 0.039 ± 0.01 mm^2^ (Table 1). After the deployment of a second stent, the pore density was increased by 45 ± 12 mm^–2^, and the porosity and pore size decreased, respectively, by 27 ± 3.2% and 0.026 ± 0.009 mm^2^ (all *p* < 0.001). See Figure 3 for the boxplots of all measured parameters corresponding to different treatment scenarios.

**TABLE 1:** Absolute and normalised parameters of stent microstructure and IA haemodynamics before and after treatments with single or dual stents (*n* = 15).

	Stent Microstructure			IA Haemodynamics																	
		
	Porosity (%)	Pore density (1/mm^2^)	Pore size (mm^2^)	IR (kg⋅m^3^/s)		EL (kg⋅m^2^/s^3^)		PD (m^2^/s^–2^)		aVEL (m/s)		aVOR (1/s)		aWSS (m^2^/s^–2^)		Mvel (m/s)		mVOR (1/s)		mWSS (m^2^/s^–2^)	
													
				[10^–3^]	%	[10^–6^]	%	[10^–2^]	%	[10^–2^]	%	[10^3^]	%	[10^–3^]	%	[10^–2^]	%	[10^3^]	%	[10^–3^]	%
Untreated condition	100	0	—	1.9 (1.4)	—	56.9 (42.2)	—	58.6 (43.2)	—	9.2 (5.5)	—	0.5 (0.3)	—	2.5 (1.7)	—	41.5 (12.5)	—	4.3 (2.3)	—	13.7 (7.2)	—
Single-stent treatment	71 (2)	20.3 (5.2)	0.039 (0.01)	1.0 (0.7)	49 (12)	10.8 (9.7)	21 (15)	67.9 (45.9)	117 (14)	2.9 (2.4)	28 (12)	0.2 (0.1)	27 (11)	0.7 (0.6)	26 (10)	33.6 (21.3)	78 (40)	9.9 (10.3)	256 (256)	31.8 (37.2)	251 (261)
Dual-stent treatment	44 (4)	65.3 (16.4)	0.013 (0.007)	0.7 (0.5)	35 (9)	6.4 (6.3)	12 (9)	81.6 (59.5)	139 (43)	1.7 (1.3)	16 (6)	0.1 (0.1)	17 (6)	0.4 (0.4)	16 (5)	38.7 (27.5)	92 (59)	8.9 (8.3)	230 (226)	26.5 (26.0)	207 (198)
*p*-value	**All *p* < 0.001^∗^**			**0.001^∗^**		0.056		0.070		**0.002^∗^**		**0.005^∗^**		**0.002^∗^**		0.453		0.770		0.609	

Values in parentheses are standard deviations. The normalised haemodynamic parameters are in relation to the respective untreated conditions. The *p*-values are results from independent two-sided *t*-tests between the single- and dual-stent treatments.

*statistical significance confirmed (*p* < 0.005).

Attributable to the second stent: (i) the IR and EL were further reduced by 14 ± 11% (*p* = 0.001) and 9 ± 12% (*p* = 0.056), in contrast to the PD, which was increased by 22 ± 32% (*p* = 0.070); and (ii) the intra-aneurysmal aVEL, aVOR, and aWSS were each further decreased by around 10% (all *p* < 0.005), in contrast to the mVEL, which was increased by 14 ± 50% (*p* = 0.453). (All values are mean ± SD, relative to the respective untreated condition.) We observed that deviations in the maximal haemodynamic parameters were much greater than those in the averaged ones, as maximal parameters are sensitive to local vascular geometries, especially after the insertion of a stent.

The greatest and the poorest reductions of the IR were observed in Case 1 (21%) and Case 9 (67%) after deployment of a single stent, and in Case 1 (13%) and Case 6 (49%) after deployment of dual stents (all values are relative to the respective untreated condition), suggesting that the flow diversion achievable depends strongly upon the geometry of the recipient artery and the location of aneurysm occurrence.

### Comparison Between Single-Stent Treatments With Devices of Different Sizes

Between the single-stent treatments that were carried out with devices of two different sizes for each aneurysm, differences in the pore density and pore size were statistically significant (both *p* < 0.001). However, this did not lead to a statistically significant difference in the post-treatment IA haemodynamics, except for the reduction in PD (*p* < 0.001), with the largest difference being 13.8%. The differences in aVOR reduction were suggestive of statistical significance (*p* = 0.028), with the largest difference being 5.3% (Table 2).

**TABLE 2:** Comparisons between single-stent treatments with devices of different diameters (scenarios “F” and “L”) in terms of stent microstructure and aneurysmal haemodynamics (*n* = 15).

Parameter	Comparison between single-stent treatments	
	
	Largest difference	*p*-values
**Microstructure**		
Porosity (%)	1.02	0.36
Pore density (mm^–2^)	1.21	<0.001*
Pore size (mm^2^)	0.003	<0.001*
**Haemodynamics (normalised to the untreated condition)**		
IR (%)	5.3	0.15
EL (%)	7.8	0.86
PD (%)	13.8	<0.001*
aVEL (%)	3.7	0.23
aVOR (%)	5.3	**0.028^†^**
aWSS (%)	6.2	0.33
mVEL (%)	89.8	0.72
mVOR (%)	903.2	0.49
mWSS (%)	819.3	0.19

*statistical significance confirmed (*p* < 0.005).

*^†^suggestive of statistical significance (*p* < 0.05).*

**p*-values in bold indicate that results of the corresponding statistical tests are suggestive of statistical significance.*

### Sizing Effects of the Earlier- and the Later-Deployed Stent in Dual-Stent Treatments

Sizing effects of the earlier-deployed stent on the final IA haemodynamics were found to be statistically significant, in terms of the IR (*p* = 0.002), EL (*p* < 0.001), and PD (*p* < 0.001), respectively, with the largest differences between treatments using first stents of different sizes being 6.9, 11.1, and 13.8% (Table 3i). Conversely, sizing effects of the later-deployed stent were found to be minor, with differences that are indicative of statistical significance only observed in the PD reduction (*p* = 0.004, largest difference of 2.1%) and the IR (*p* = 0.012, largest difference of 1.4%) (Table 3ii and Figure 2).

**TABLE 3:** Sizing effects of (i) the earlier-deployed stent and (ii) the later-deployed stent on the final stent microstructure and aneurysmal haemodynamics after dual-stent treatments (*n* = 15).

	Comparisons between dual-stent treatments					
	
Parameter	(i) Sizing effects of the earlier-deployed stent		(ii) Sizing effects of the later-deployed stent			
		
	(“FinF” and “LinF”) *vs.* (“FinL” and “LinL”)		(“FinF” *vs.* “LinF”)		(“FinL” *vs.* “LinL”)	
			
	Largest differences	*p*-values	Largest differences	*p*-values	Largest differences	*p*-values
**Microstructure**						
Porosity (%)	5.13	0.72	1.63	**0.009**^†^	1.55	0.32
Pore density (mm^–2^)	28.26	0.29	10.95	0.002*	7.02	0.31
Pore size (mm^2^)	0.009	0.35	0.003	0.003*	0.003	0.54
**Haemodynamics (normalised to the untreated condition)**						
IR (%)	6.9	0.002*	1.0	0.13	1.4	**0.012**^†^
EL (%)	11.1	<0.001*	0.5	0.64	1.1	0.34
PD (%)	13.8	<0.001*	0.7	0.004*	2.1	<0.001*
aVEL (%)	6.6	0.13	0.8	0.14	1.1	0.17
aVOR (%)	6.7	0.19	1.1	0.58	1.2	0.65
aWSS (%)	6.9	**0.046**^†^	1.1	0.60	1.2	0.74
mVEL (%)	183.7	0.45	39.7	0.76	47.9	0.37
mVOR (%)	770.6	0.84	191.8	0.17	156.9	0.19
mWSS (%)	677.0	0.33	167.3	0.26	140.0	0.08

Pearson χ^2^ tests were used for comparisons in group i and paired-sample *t*-tests were used for comparisons in group ii.

*statistical significance confirmed (*p* < 0.005).

*^†^suggestive of statistical significance (*p* < 0.05).*

*p-values in bold indicate that results of the corresponding statistical tests are suggestive of statistical significance.*

### Correlation Between Stent Configurations and the Post-treatment IA Haemodynamics

**FIGURE 4:**
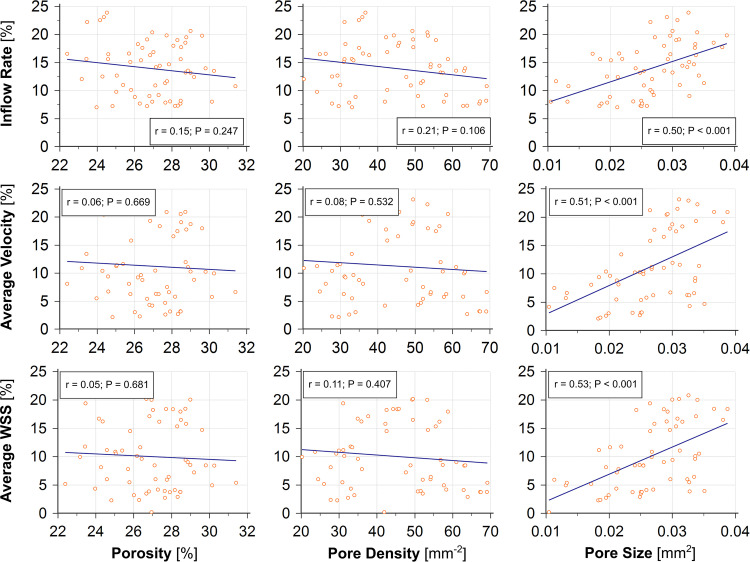
The change in pore size **(right)** caused by deployment of a second stent shows good correlations (*r* ≥ 0.50, all *p* < 0.001) with reductions of the post-treatment IR, aVEL, and aWSS, compared with the changes in porosity (**left**, *r* < 0.15, all *p* > 0.24) and pore density (middle, *r* ≤ 0.21, all *p* > 0.10).

In comparison to porosity and pore density, we found that the changes in average pore size between stent wires at the aneurysm orifice had a stronger correlation with reductions in most of the investigated haemodynamic parameters — IR (*r* = 0.50), PD (*r* = 0.63), aVEL (*r* = 0.51), aVOR (*r* = 0.52), and aWSS (*r* = 0.53) (*n* = 60, all *p* < 0.001). Reductions in the remaining haemodynamic parameters (EL and all maximal parameters) were all very weakly correlated with changes in each of the three stent morphological parameters (Table 4 and Figure 4).

**TABLE 4:** Correlations between changes in the stent microstructural parameters and reductions in the normalised aneurysmal haemodynamic parameters after the deployment of a second stent (*n* = 60).

	Inflow Rate	Energy Loss	Pressure Drop	Average			Maximal		
					
				Velocity	Vorticity	WSS	Velocity	Vorticity	WSS
Porosity	*r* = −0.15 (−0.39, 0.11) *p* = 0.25	*r* = −0.01 (−0.26, 0.25) *p* = 0.94	***r* = −0.33** (−0.54, -0.08) *p* = 0.010*	*r* = −0.06 (−0.31, 0.20) *p* = 0.67	*r* = −0.04 (−0.29, 0.21) *p* = 0.74	*r* = −0.05 (−0.30, 0.20) *p* = 0.68	*r* = 0.12 (−0.14, 0.36) *p* = 0.37	*r* = 0.15 (−0.11, 0.39) *p* = 0.24	*r* = 0.14 (−0.11, 0.38) *p* = 0.27
Pore density	*r* = 0.21 (−0.05, 0.44) *p* = 0.11	*r* = −0.06 (−0.31, 0.20) *p* = 0.67	***r* = 0.32** (0.07, 0.53) *p* = 0.012*	*r* = 0.08 (−0.18, 0.33) *p* = 0.53	*r* = 0.10 (−0.16, 0.34) *p* = 0.45	*r* = 0.11 (−0.15, 0.35) *p* = 0.41	*r* = 0.01 (−0.25, 0.26) *p* = 0.95	*r* = 0.05 (−0.20, 0.30) *p* = 0.68	*r* = 0.07 (−0.19, 0.32) *p* = 0.59
Pore size	***r* = 0.50** (0.28, 0.67) ***p* < 0.001**^†^	*r* = 0.06 (−0.19, 0.31) *p* = 0.63	***r* = 0.63** (0.44, 0.76) ***p* < 0.001**^†^	***r* = 0.51** (0.29, 0.68) ***p* < 0.001**^†^	***r* = 0.52** (0.30, 0.68) ***p* < 0.001**^†^	***r* = 0.53** (0.32, 0.69) ***p* < 0.001**^†^	*r* = −0.11 (−0.36, 0.14) *p* = 0.38	*r* = 0.03 (−0.23, 0.28) *p* = 0.82	*r* = 0.01 (−0.24, 0.26) *p* = 0.94

Values in parentheses indicate the 95% confidence intervals of the corresponding Pearson correlation coefficient, *r*.

*statistical significance confirmed (*p* < 0.005).

*^†^suggestive of statistical significance (*p* < 0.05).*

*p-values in bold indicate that results of the corresponding statistical tests are suggestive of statistical significance.*

## Discussion

### Stent Pore Size May Be Used to Predict a Favourable Haemodynamic Outcome

The average pore size in stent wires spanning the aneurysm orifice is a morphological parameter that has been used less commonly than porosity or pore density in the haemodynamic analysis of flow-diversion treatments. Despite the fact that the three parameters are related to each other, we found that the average pore size was the only microstructural parameter demonstrating consistently good correlations with major haemodynamic parameters used in this study to assess the flow-diversion outcomes — e.g., the IR, PD, aVEL, aVOR, and aWSS (*n* = 60 for each correlation analysis, all *r >* 0.5, and all *p* < 0.001).

**FIGURE 5:**
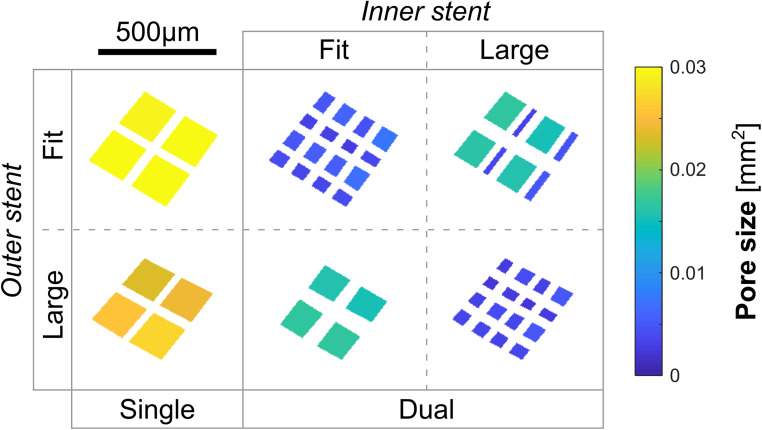
Projections of pores in a representative region of the FD stent in the microstructural analysis of case ID C0016. When strands of the earlier- and the later-deployed stents were partially overlapped (refer to the middle image in the bottom row), the pore size could still be decreased by around 30%, while the number of pores (*n* = 4) within the region of interest (pore density) remains unchanged.

In dual-stent treatments, complete or partial overlap of stent braids is sometimes observed, especially when the two stents are of identical nominal diameter, causing a smaller gain in the final metal coverage across the aneurysm orifice (Shapiro et al., 2014). When stent braids are partially overlapped, whilst additional resistance to the aneurysmal inflow could still be attained, the local pore density would not change, as the overlapped stent braids would not yield new pores. In contrast, the stent porosity and pore size would be decreased, with the change in the latter being greater than that in the former. See Figure 5 for a demonstration of the local pore size measurement in such a circumstance. Stent pore size is a surrogate measure for the local hydraulic diameter, which immediately determines the resistance to flow passing through the wire mesh and entering the aneurysm sac. For this reason, out of the three microstructural parameters, changes in the stent pore size exhibited the best correlation with IA haemodynamics, and demonstrated its potential as an index for predicting the treatment outcomes.

In addition, we noticed that when the earlier-deployed stents were of a smaller diameter, differences in the final stent porosity (*p* < 0.01), pore density (*p* = 0.002), and pore size (*p* = 0.003) were indicative of statistical significance (Table 3ii). This is because when a stent of a larger diameter was deployed into a smaller one, wires of the larger stent would expand substantially less than the nominal diameter — where the specified porosity and pore density could be attained (United States Food and Drug Administration, 2011). This would markedly affect the final structure of devices deployed in a dual-stent treatment.

### Substantial Sizing Effect From the Earlier-Deployed Stent in Dual-Stent Treatments

We observed that size of the earlier-deployed stent had statistically significant effects on the final flow reduction achievable through dual-stent implantation. The underlying mechanism is that the diameter of the outer-layer stent would predominantly determine how the haemodynamics in the parent artery would be modified, in terms of changes to the local flow patterns and resistance to the blood flow over the aneurysm segment, etc.

Simple theories of fluid mechanics indicate that a stent of a smaller diameter would yield a greater resistance to the flow passing through its lumen, causing a larger pressure drop across the stented vascular segment (Rubenstein et al., 2015). This phenomenon was observed in all simulated treatment scenarios. Coupled with the unique vascular geometry of a patient, the diameter of the first stent would then determine the location, strength, and angle of the aneurysmal inflow after stent deployment, thereby affecting the final IA haemodynamics. Conversely, insertion of a second device following a standard deployment procedure [i.e., deployment without a push-and-pull (Ma et al., 2014) manoeuvre] would primarily enhance the density of stent wires, rather than introduce drastic changes to the vascular lumen already formed by the first stent. Therefore, sizing effects from the later-deployed stent were found to be smaller, as only limited alterations were made to the configuration of aneurysmal inflow (see Figure 2, for instance).

Furthermore, appropriate choice of the earlier-deployed stent would also contribute to firm wall apposition, which reportedly is an important factor associated with stent endothelialisation and aneurysm occlusion (Rouchaud et al., 2016). Inappropriate choice of the first stent, however, may inadvertently result in a gap between the stent and the arterial wall, through which a jet of bypass flow may enter the aneurysm, impinging on the aneurysm wall (Chong et al., 2014). To assist in identifying the optimal stent size for a specific patient, as demonstrated in the present study as well as in other relevant works (Zhang et al., 2017a; Chong et al., 2019), virtual stenting and the subsequent CFD analysis may be a useful tool for clinicians to examine the stent structure and assess the haemodynamic outcomes prior to a real treatment.

### Limitations

For the stent deployment simulation, we simplified the interaction between the stent wires and the arterial wall, as well as the interaction between the earlier- and the later-deployed stents, assuming a stent to be a rigid entity that would not deform once it is completely deployed. Further limitation lies in the rigid-wall assumption, which neglects the vascular deformation due to fluid flow. This is because to model those interactions would require relevant material properties for the vascular wall and the stent strand to be accurately characterised, and incorporating them into the stent deployment simulation can be very resource-demanding. Although the present settings for virtual stent deployment are commonly adopted with reasonable justifications (Damiano et al., 2015, 2017), its appropriateness deserves future investigation as technical progress in resolving vascular physiopathology is recently being made (Bianchi et al., 2017).

In addition, it should be noted that the results of stent deployment may be of great uncertainty, as the process of stent deployment depends substantially upon the predilection of the treating clinicians, in terms of the landing positions, unsheathing process, and the pathway along which a stent is released. It would be a promising direction for future study to look into the effects of those factors on the resulting stent micro-structures and their associated aneurysmal haemodynamics.

Moreover, future work would benefit from collecting patient-specific vascular boundary conditions and adopting them in CFD analysis, which would allow transient flow simulation to better reproduce physiologically realistic aneurysmal haemodynamics over an entire cardiac cycle. That way, time-dependent haemodynamic parameters, such as the space-time patterns of WSS (Gizzi et al., 2011; Morbiducci et al., 2015; Nestola et al., 2015) and SR (Ribeiro de Sousa et al., 2016), can then be calculated to examine their correlation with clinical treatment outcomes.

## Conclusion

Three microstructural parameters of FD stents and nine IA haemodynamic parameters were quantified and systematically compared across 15 patients after single- and dual-stent treatments using devices of different sizes.

We found that:

i. deployment of a second stent further reduced the aneurysmal inflow rate (IR) and energy loss (EL) by 14 ± 11% (*p* = 0.001) and 9 ± 12% (*p* = 0.056), relative to the untreated condition;
ii. different diameters of the earlier-deployed stent led to largest differences of 6.9% for the final IR reduction, and 11.1% for the EL reduction; whereas sizing effects from the later-deployed stent were minor (≤2.1 %); and
iii. the change in stent pore size was the only microstructural parameter demonstrating a strong correlation with the reduction in the post-treatment aneurysmal haemodynamics, in terms of the IR (*r* = 0.50, *p <* 0.001) and pressure drop (*r* = 0.63, *p <* 0.001).

The size of the earlier-deployed stent has a significant impact on the IA haemodynamics after dual-stent treatment. The average pore size of stent wires at the aneurysm orifice shows promise as a potential index for predicting the efficacy of flow-diversion treatment.

## Data Availability Statement

The raw data supporting the conclusions of this article will be made available by the authors, without undue reservation.

## Ethics Statement

Ethical review and approval was not required for the study on human participants in accordance with the local legislation and institutional requirements. Written informed consent for participation was not required for this study in accordance with the national legislation and the institutional requirements.

## Author Contributions

MZ and ST: guarantors of integrity of entire study, designed computational and analysis protocol, and wrote the manuscript. MZ: original concept. MZ, MO, and ST: collected patients’ data. MZ, ST, and YL: performed computational experiments. All authors analysed the data, revised manuscript for important intellectual content, approved final version of submitted manuscript, and agreed to ensure any questions related to the work are appropriately resolved.

## Conflict of Interest

The authors declare that the research was conducted in the absence of any commercial or financial relationships that could be construed as a potential conflict of interest.

## Abbreviations

Avel: average intra-aneurysmal velocity
Avor: average intra-aneurysmal vorticity
aWSS: average aneurysmal wall shear stress
CFD: computational fluid dynamics
EL: energy loss
IA: intracranial aneurysm
IR: inflow rate
mVEL: maximal intra-aneurysmal velocity
mVOR: maximal intra-aneurysmal vorticity
mWSS: maximal aneurysmal wall shear stress.
