# Supplementary material for: Association Between Aneurysmal Haemodynamics and Device Microstructural Characteristics After Flow-Diversion Treatments With Dual Stents of Different Sizes: A Numerical Study
Source: Front Physiol. 2021 May 25;12:663668. doi: 10.3389/fphys.2021.663668 (PMC8185279; doi:10.3389/fphys.2021.663668)
Supplement: Supplementary file 1 [file Data_Sheet_1.docx]

# **Online Table 1**

**Online Table 1.** Patient demographics, aneurysm morphology, and the ICA inlet volumetric flowrates deduced from the power-law estimation.

| No. | Case ID^*^ | Gender | Age | Aspect  ratio | Dome-to-neck ratio | Neck width (mm) | Proximal diameter (mm) | Inlet VFR (mL/s) | FD diameter (mm) | |
| --- | --- | --- | --- | --- | --- | --- | --- | --- | --- | --- |
|  |  |  |  |  |  |  |  |  | ‘F’ | ‘L’ |
| 1 | C0005 | F | 26 | 1.50 | 1.42 | 2.72 | 2.18 | 1.008 | 2.75 | 3.00 |
| 2 | C0006 | F | 45 | 0.74 | 1.13 | 7.57 | 3.38 | 2.422 | 3.25 | 3.50 |
| 3 | C0013 | F | 85 | 1.30 | 0.83 | 9.34 | 4.76 | 4.803 | 4.75 | 5.00 |
| 4 | C0016 | F | 42 | 1.45 | 1.33 | 5.37 | 2.58 | 1.411 | 2.75 | 3.00 |
| 5 | C0024 | F | 35 | 0.93 | 1.33 | 6.51 | 3.62 | 2.778 | 3.50 | 3.75 |
| 6 | C0026 | F | 42 | 1.54 | 1.29 | 6.43 | 4.76 | 4.803 | 4.75 | 5.00 |
| 7 | C0034 | F | 42 | 0.93 | 1.16 | 7.31 | 3.42 | 2.480 | 3.25 | 3.50 |
| 8 | C0035 | F | 57 | 1.98 | 1.67 | 7.02 | 3.40 | 2.451 | 3.25 | 3.50 |
| 9 | C0041 | F | 73 | 0.64 | 1.18 | 3.54 | 4.82 | 4.925 | 4.75 | 5.00 |
| 10 | C0042 | F | 45 | 1.76 | 1.29 | 7.36 | 3.06 | 1.985 | 3.25 | 3.50 |
| 11 | C0065 | M | 47 | 1.68 | 1.77 | 2.61 | 2.38 | 1.201 | 2.25 | 2.50 |
| 12 | C0067 | F | 42 | 1.30 | 1.17 | 3.63 | 2.92 | 1.808 | 3.50 | 3.75 |
| 13 | C0089 | F | 48 | 0.93 | 0.99 | 5.90 | 4.20 | 3.740 | 4.00 | 4.25 |
| 14 | C0090 | F | 42 | 0.82 | 1.40 | 9.25 | 4.18 | 3.704 | 4.25 | 4.50 |
| 15 | C0097 | F | 84 | 1.60 | 1.13 | 5.12 | 2.88 | 1.758 | 3.50 | 3.75 |
| Mean (SD) | | M: 1/15 | 50.3 (16.6) | 1.27 (0.40) | 1.27 (0.23) | 5.98 (2.07) | 3.50 (0.84) | 2.752 (1.291) | 3.58 (0.75) | 3.83 (0.75) |

^*^Case ID refers to the original case number in the *Aneurisk* repository.

VFR: volumetric flowrate; and FD: flow diverter.

# **Online Table 2**

**Online Table 2**. Means, medians, and standard deviations of the morphological and haemodynamic parameters calculated for the 15 patient aneurysms.

| **Parameters** | **Units** | **NT** | | | **F** | | | **L** | | | **FinF** | | |
| --- | --- | --- | --- | --- | --- | --- | --- | --- | --- | --- | --- | --- | --- |
|  |  | *Mean* | *Median* | *SD* | *Mean* | *Median* | *SD* | *Mean* | *Median* | *SD* | *Mean* | *Median* | *SD* |
| **Porosity** | (%) | — | — | — | 7.1E-01 | 7.1E-01 | 2.3E-02 | 7.1E-01 | 7.1E-01 | 2.5E-02 | 4.3E-01 | 4.2E-01 | 3.7E-02 |
| **Pore density** | (mm^−2^) | — | — | — | 2.1E+01 | 2.1E+01 | 5.1E+00 | 2.0E+01 | 2.0E+01 | 5.5E+00 | 7.0E+01 | 7.4E+01 | 1.7E+01 |
| **Pore size** | (mm^2^) | — | — | — | 3.8E-02 | 3.6E-02 | 9.9E-03 | 4.0E-02 | 3.8E-02 | 1.1E-02 | 1.1E-02 | 7.9E-03 | 6.4E-03 |
| **IR** | (kg·m^3^/s) | 1.9E-03 | 1.6E-03 | 1.4E-03 | 9.7E-04 | 8.7E-04 | 7.6E-04 | 9.6E-04 | 8.4E-04 | 7.5E-04 | 6.8E-04 | 6.9E-04 | 5.2E-04 |
| **EL** | (kg·m^2^/s^3^) | 5.7E-05 | 4.5E-05 | 4.2E-05 | 1.0E-05 | 1.0E-05 | 9.7E-06 | 1.1E-05 | 1.0E-05 | 1.0E-05 | 6.1E-06 | 4.3E-06 | 6.4E-06 |
| **PD** | (m^2^/s^˗2^) | 5.9E-01 | 4.8E-01 | 4.3E-01 | 6.9E-01 | 5.5E-01 | 4.7E-01 | 6.7E-01 | 5.4E-01 | 4.7E-01 | 8.3E-01 | 5.8E-01 | 6.2E-01 |
| **aVEL** | (m/s) | 9.2E-02 | 8.2E-02 | 5.5E-02 | 2.9E-02 | 2.2E-02 | 2.4E-02 | 2.9E-02 | 1.8E-02 | 2.5E-02 | 1.6E-02 | 1.2E-02 | 1.3E-02 |
| **aVOR** | (1/s) | 5.5E+02 | 4.2E+02 | 3.4E+02 | 1.7E+02 | 1.3E+02 | 1.5E+02 | 1.6E+02 | 1.0E+02 | 1.5E+02 | 9.8E+01 | 6.5E+01 | 8.2E+01 |
| **aWSS** | (m^2^/s^˗2^) | 2.5E-03 | 1.7E-03 | 1.7E-03 | 7.2E-04 | 5.1E-04 | 6.3E-04 | 7.2E-04 | 4.3E-04 | 6.9E-04 | 4.2E-04 | 2.7E-04 | 3.5E-04 |
| **mVEL** | (m/s) | 4.1E-01 | 4.1E-01 | 1.3E-01 | 3.4E-01 | 3.6E-01 | 2.0E-01 | 3.3E-01 | 2.3E-01 | 2.4E-01 | 4.1E-01 | 4.2E-01 | 3.1E-01 |
| **mVOR** | (1/s) | 4.3E+03 | 3.9E+03 | 2.3E+03 | 1.2E+04 | 7.8E+03 | 1.2E+04 | 7.9E+03 | 3.7E+03 | 8.7E+03 | 1.0E+04 | 1.0E+04 | 8.2E+03 |
| **mWSS** | (m^2^/s^˗2^) | 1.4E-02 | 1.2E-02 | 7.2E-03 | 4.2E-02 | 3.1E-02 | 4.4E-02 | 2.2E-02 | 1.1E-02 | 2.6E-02 | 3.0E-02 | 2.5E-02 | 2.6E-02 |

Note: NT: untreated condition; F: treatment with a single stent of ‘fit’ size; L: treatment with a single stent of ‘larger’ size; and FinL: dual-stent treatment with the later-deployed stent being of ‘fit’ size and the earlier-deployed stent being of ‘larger’ size. The same naming convention applies to the remaining treatment scenarios: FinF, LinF, and LinL.

| *Continuation of Online Table 2* | | | | | | | | | |
| --- | --- | --- | --- | --- | --- | --- | --- | --- | --- |
|  | **FinL** | | | **LinF** | | | **LinL** | | |
|  | *Mean* | *Median* | *SD* | *Mean* | *Median* | *SD* | *Mean* | *Median* | *SD* |
| **Porosity** | 4.4E-01 | 4.5E-01 | 3.5E-02 | 4.4E-01 | 4.4E-01 | 3.4E-02 | 4.4E-01 | 4.3E-01 | 4.5E-02 |
| **Pore density** | 6.4E+01 | 6.7E+01 | 1.4E+01 | 6.0E+01 | 5.6E+01 | 1.4E+01 | 6.7E+01 | 7.0E+01 | 2.0E+01 |
| **Pore size** | 1.3E-02 | 1.1E-02 | 5.9E-03 | 1.4E-02 | 1.2E-02 | 6.3E-03 | 1.2E-02 | 8.1E-03 | 8.5E-03 |
| **IR** | 6.7E-04 | 6.5E-04 | 5.2E-04 | 7.0E-04 | 7.0E-04 | 5.4E-04 | 6.4E-04 | 6.6E-04 | 4.9E-04 |
| **EL** | 7.1E-06 | 5.8E-06 | 7.2E-06 | 6.0E-06 | 4.4E-06 | 6.2E-06 | 6.6E-06 | 5.7E-06 | 6.2E-06 |
| **PD** | 8.1E-01 | 5.7E-01 | 6.0E-01 | 8.3E-01 | 5.8E-01 | 6.2E-01 | 8.0E-01 | 5.6E-01 | 6.0E-01 |
| **aVEL** | 1.7E-02 | 1.0E-02 | 1.5E-02 | 1.7E-02 | 1.3E-02 | 1.3E-02 | 1.6E-02 | 1.1E-02 | 1.4E-02 |
| **aVOR** | 9.8E+01 | 5.6E+01 | 8.8E+01 | 9.8E+01 | 6.6E+01 | 7.5E+01 | 9.6E+01 | 5.8E+01 | 8.7E+01 |
| **aWSS** | 4.2E-04 | 2.3E-04 | 3.8E-04 | 4.1E-04 | 2.8E-04 | 3.2E-04 | 4.1E-04 | 2.4E-04 | 3.8E-04 |
| **mVEL** | 3.3E-01 | 2.2E-01 | 2.8E-01 | 4.1E-01 | 4.1E-01 | 2.9E-01 | 4.0E-01 | 4.4E-01 | 2.3E-01 |
| **mVOR** | 6.7E+03 | 4.1E+03 | 8.0E+03 | 8.5E+03 | 4.4E+03 | 9.3E+03 | 1.0E+04 | 7.2E+03 | 8.1E+03 |
| **mWSS** | 1.8E-02 | 9.7E-03 | 2.1E-02 | 2.6E-02 | 1.3E-02 | 2.7E-02 | 3.2E-02 | 2.5E-02 | 3.0E-02 |

# **Online Figure 1**


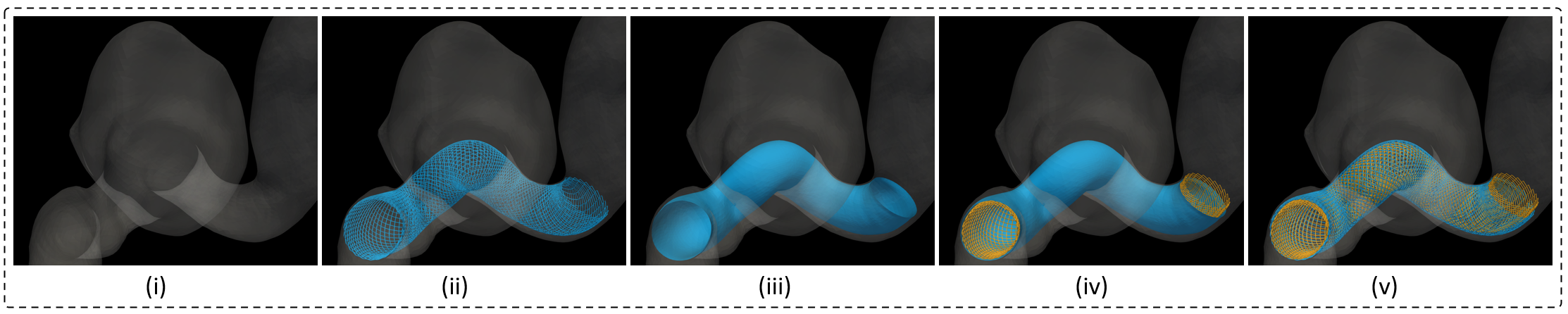


**Online Figure 1.** Schematic depiction of the virtual deployment of dual stents. (i) the aneurysm geometry (untreated condition); (ii) deployment of the first stent; (iii) surface fitting of the first-deployed stent; (iv) deployment of the second stent into the pseudo-vascular lumen of the first stent; and (v) assembly of the earlier- and the later-deployed stents.

# **Online Figure 2**


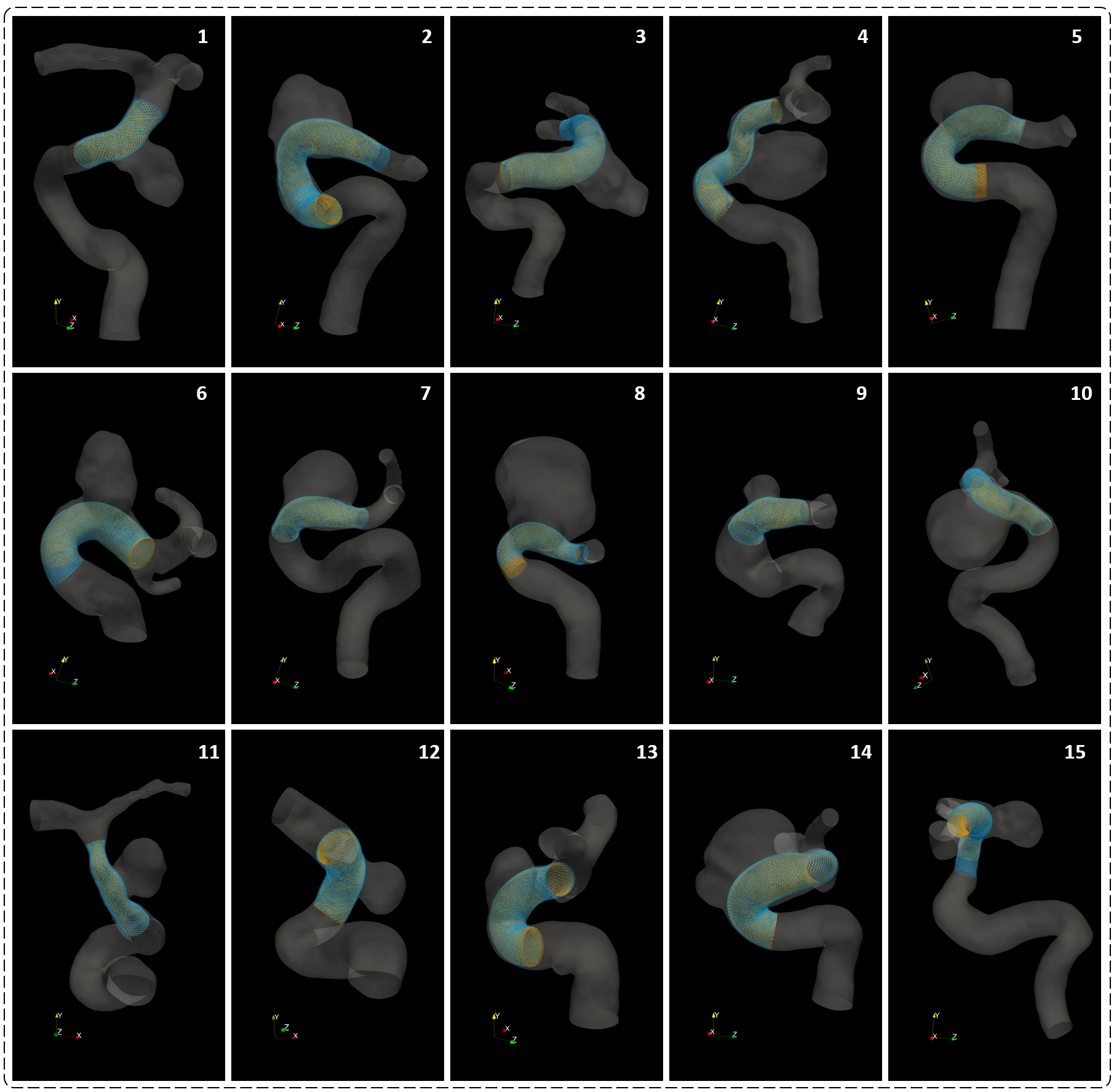


**Online Figure 2.** Morphology of the IA geometries included in this study and demonstration of the virtual dual-stent treatments corresponding to scenarios ‘FinF’ for each case. Row 1: Cases 1–5; Row 2: Cases 6–10; and Row 3: Cases 11–15. FD stents in blue represent the earlier-deployed stent; and FD stents in yellow represent the later-deployed stents.

# **Supplementary Material**

This supplementary material provides information on the strategy used in the present study for deploying multiple FD stents.

The PED is a flexible and self-expandable device braided from 48 metal wires, with different combinations of device length (10–35 mm) and diameter (2.5–5 mm) available on the market. We employed a recently reported and validated spring–mass model to represent the topology and simulate the deployment of a PED stent. (Spranger and Ventikos, 2014; Peach et al., 2016, 2017)

In the spring–mass model, the intersection of any two stent wires is simplified as a mass point connected to neighbouring mass points by fictitious springs. Using this strategy, the structure of a PED stent could be represented by a collection of springs and mass points. The internal forces of the FD stent could therefore be estimated by calculating the restoring forces of each spring using Hooke’s law:

$$\boldsymbol{F}_{\boldsymbol{i}}=\sum_{j=1}^{n_{i}} k_{ij}\left( \boldsymbol{\delta}_{\boldsymbol{j}}-\boldsymbol{\delta}_{\boldsymbol{i}} \right), \left( 1 \right)$$

in which $\boldsymbol{\delta}_{\boldsymbol{i}}$ denotes the displacement of node $i$; $k_{ij}$ stands for the stiffness of the fictitious spring connecting node $i$ and its neighbor $j$; and $n_{i}$ is the number of nodes directly connected to node $i$. The stiffness of a spring was correlated with the wire length and thickness being represented.

For the simulation of stent deployment, the FD stent was assumed to first be crimped in alignment with the centreline of the recipient artery, and then to expand to its unloaded condition, driven by the internal restoring forces. In the restoring process, a contact detection algorithm was employed to control the stent nodal movements within the vascular boundaries.(Zhang et al., 2017)

For the deployment of a single stent, the boundary constraint was only the vascular wall. For the deployment of a second stent, we first performed a surface fitting of the fully expanded vertices of the earlier-deployed stent, and then set the fitted surface (treated as a vascular lumen), along with the vascular wall, as the boundary constraints for the deployment of the second stent (see Figure S1 for the schematic). After the fully expanded status was reached, we finally converted the FD nodal information to a FD model by sweeping a circular cross-section through the connected fictitious springs using Paraview.(Henderson et al., 2004)


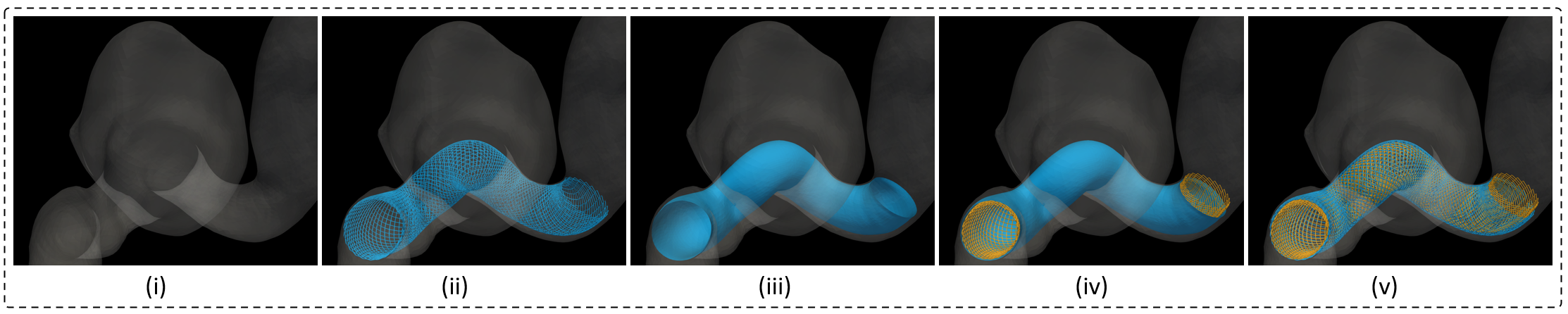


**Figure S1.** Schematic for the virtual deployment of dual stents. (i) the aneurysm geometry (untreated condition); (ii) deployment of the first stent; (iii) surface fitting of the first-deployed stent; (iv) deployment of the second stent into the pseudo vascular lumen of the first stent; and (v) assembly of the earlier- and the later-deployed stents.

**References:**

Henderson, A., Ahrens, J., and Law, C. (2004). *The ParaView Guide*. Kitware Clifton Park, NY.

Peach, T., Spranger, K., and Ventikos, Y. (2017). Virtual flow-diverter treatment planning: The effect of device placement on bifurcation aneurysm haemodynamics. *Proc Inst Mech Eng H* 231, 432–443. doi:10.1177/0954411916673674.

Peach, T. W., Spranger, K., and Ventikos, Y. (2016). Towards Predicting Patient-Specific Flow-Diverter Treatment Outcomes for Bifurcation Aneurysms: From Implantation Rehearsal to Virtual Angiograms. *Ann Biomed Eng* 44, 99–111. doi:10.1007/s10439-015-1395-3.

Spranger, K., and Ventikos, Y. (2014). Which Spring is the Best? Comparison of Methods for Virtual Stenting. *IEEE Transactions on Biomedical Engineering* 61, 1998–2010. doi:10.1109/TBME.2014.2311856.

Zhang, M., Li, Y., Zhao, X., Verrelli, D. I., Chong, W., Ohta, M., et al. (2017). Haemodynamic effects of stent diameter and compaction ratio on flow-diversion treatment of intracranial aneurysms: A numerical study of a successful and an unsuccessful case. *Journal of Biomechanics* 58, 179–186. doi:10.1016/j.jbiomech.2017.05.001.
